# Bi-allelic *UGGT1* variants cause a congenital disorder of glycosylation

**DOI:** 10.1016/j.ajhg.2025.03.018

**Published:** 2025-04-22

**Authors:** Zain Dardas, Laura Harrold, Daniel G. Calame, Claire G. Salter, Takashi Kikuma, Kevin P. Guay, Bobby G. Ng, Kanae Sano, Ahmad K. Saad, Haowei Du, Riccardo Sangermano, Sohil G. Patankar, Shalini N. Jhangiani, Semra Gürsoy, Mohamed S. Abdel-Hamid, Mahmoud K.H. Ahmed, Reza Maroofian, Rauan Kaiyrzhanov, Kamran Salayev, Wendy D. Jones, Ana Pérez Caballero, Lucy McGavin, Michael Spiller, Miranda Durkie, Nick Wood, Lauren O’Grady, Paula Goldenberg, Ann M. Neumeyer, Amber Begtrup, Sherif F. Abdel-Ghafar, Maha S. Zaki, Hilde Van Esch, Jennifer E. Posey, Olivia K. Wenger, Ethan M. Scott, Kinga M. Bujakowska, Richard A. Gibbs, Davut Pehlivan, Dana Marafi, Joseph S. Leslie, Nishanka Ubeyratna, Jacob Day, Martina Owens, Jessica Settle, Soher Balkhy, Abdullah Tamim, Lama Alabdi, Fowzan S. Alkuraya, Yoichi Takeda, Hudson H. Freeze, Daniel N. Hebert, James R. Lupski, Andrew H. Crosby, Emma L. Baple

**Affiliations:** 1 Department of Molecular and Human Genetics, Baylor College of Medicine, Houston, TX, USA; 2 Department of Clinical and Biomedical Sciences (Medical School), Faculty of Health and Life Sciences, University of Exeter, Exeter, UK; 3 Texas Children’s Hospital, Houston, TX, USA; 4 Section of Pediatric Neurology and Developmental Neuroscience, Department of Pediatrics, Baylor College of Medicine, Houston, TX, USA; 5 Wessex Clinical Genetics Service, Princess Anne Hospital, Southampton, UK; 6 Department of Biotechnology, College of Life Sciences, Ritsumeikan University, 1-1-1 Noji-Higashi, Kusatsu, Shiga 525-8577, Japan; 7 Program in Molecular and Cellular Biology, University of Massachusetts Amherst, Amherst, MA, USA; 8 Department of Biochemistry and Molecular Biology, University of Massachusetts Amherst, Amherst, MA, USA; 9 Human Genetics Program, Sanford Burnham Prebys Medical Discovery Institute, La Jolla, CA, USA; 10 Medical Molecular Genetics Department, Human Genetics and Genome Research Institute, National Research Centre, Cairo, Egypt; 11 Ocular Genomics Institute, Department of Ophthalmology, Massachusetts Eye and Ear, Harvard Medical School, Boston, MA, USA; 12 Human Genome Sequencing Center, Baylor College of Medicine, Houston, TX, USA; 13 Department of Pediatric Genetics, Dokuz Eylul University, Faculty of Medicine, Izmir, Turkey; 14 Department of Prenatal Diagnosis and Fetal Medicine, Human Genetics and Genome Research Institute, National Research Centre, Cairo, Egypt; 15 Department of Neuromuscular Diseases, UCL Queen Square Institute of Neurology, University College London, London, UK; 16 Fetal-Neonatal Neuroimaging & Developmental Science Center, Division of Newborn Medicine, Boston Children’s Hospital, Boston, MA, USA; 17 The North East Thames Regional Genetics Service, Great Ormond Street Hospital, London, UK; 18 North Thames Genomic Laboratory Hub, Great Ormond Street NHS Foundation Trust, London, UK; 19 University Hospitals Plymouth NHS Trust, Plymouth, UK; 20 University of Plymouth, Plymouth, UK; 21 Sheffield Diagnostic Genetics Service, North East and Yorkshire Genomic Laboratory Hub, Sheffield Children’s NHS Foundation Trust, Sheffield, UK; 22 Bradford Teaching Hospitals NHS Foundation Trust, Bradford, UK; 23 Massachusetts General Hospital for Children, Division of Medical Genetics and Metabolism, Boston, MA, USA; 24 Massachusetts General Hospital for Children, Lurie Center for Autism, Lexington, MA, USA; 25 GeneDx LLC, Gaithersburg, MD, USA; 26 Department of Clinical Genetics, Human Genetics and Genome Research Institute, National Research Centre, Cairo, Egypt; 27 Center for Human Genetics, University Hospitals Leuven, University of Leuven, Leuven, Belgium; 28 New Leaf Center, Clinic for Special Children, Mount Eaton, OH, USA; 29 Department of Pediatrics, Faculty of Medicine, Kuwait University, Kuwait City, Kuwait; 30 Exeter Genomics Laboratory, RILD Wellcome Wolfson Medical Research Centre, Royal Devon University Healthcare NHS Foundation Trust, Exeter, UK; 31 Department of Pediatrics, King Faisal Specialist Hospital and Research Center, Jeddah, Saudi Arabia; 32 Department of Translational Genomics, Center for Genomic Medicine, King Faisal Specialist Hospital and Research Center, Riyadh, Saudi Arabia; 33 Department of Pediatrics, Baylor College of Medicine, Houston, TX, USA; 34 Peninsula Clinical Genetics Service, Royal Devon University Healthcare NHS Foundation Trust, Gladstone Road, Exeter, UK

**Keywords:** UGGT1, UDP-glucose:glycoprotein glucosyltransferase 1, MOGS, congential disorder of glycosylation, autosomal recessive, microcephaly, neurodevelopmental disorder, N-linked glycosylation, monogenic disorder

## Abstract

Congenital disorders of glycosylation (CDGs) comprise a large heterogeneous group of metabolic conditions caused by defects in glycoprotein and glycolipid glycan assembly and remodeling, a fundamental molecular process with wide-ranging biological roles. Herein, we describe bi-allelic *UGGT1* variants in fifteen individuals from ten unrelated families of various ethnic backgrounds as a cause of a distinctive CDG of variable severity. The cardinal clinical features of UGGT1-CDG involve developmental delay, intellectual disability, seizures, characteristic facial features, and microcephaly in the majority (9/11 affected individuals for whom measurements were available). The more severely affected individuals display congenital heart malformations, variable skeletal abnormalities including scoliosis, and hepatic and renal involvement, including polycystic kidneys mimicking autosomal recessive polycystic kidney disease. Clinical studies defined genotype-phenotype correlations, showing bi-allelic *UGGT1* loss-of-function variants associated with increased disease severity, including death in infancy. *UGGT1* encodes UDP-glucose:glycoprotein glucosyltransferase 1, an enzyme critical for maintaining quality control of N-linked glycosylation. Molecular studies showed that pathogenic *UGGT1* variants impair UGGT1 glucosylation and catalytic activity, disrupt mRNA splicing, or inhibit endoplasmic reticulum (ER) retention. Collectively, our data provide a comprehensive genetic, clinical, and molecular characterization of UGGT1-CDG, broadening the spectrum of N-linked glycosylation disorders.

## Graphical abstract

**Figure:**
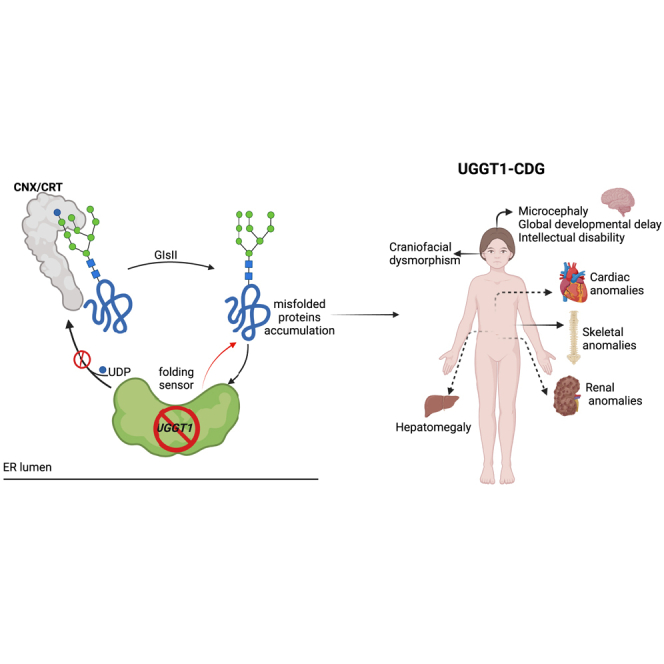

## Teaser

Bi-allelic *UGGT1* variants cause a distinct congenital disorder of glycosylation (UGGT1-CDG) with variable severity, characterized by neurodevelopmental impairment, seizures, dysmorphic features, and multiorgan involvement. Functional studies reveal diverse pathogenic mechanisms, expanding the spectrum of N-linked glycosylation disorders and providing new insights into *UGGT1*’s role in human disease.

## Introduction

Glycoconjugates are a heterogeneous repertoire of carbohydrate chains (glycans) covalently linked to proteins or lipids formed through the process of glycosylation. Glycoconjugate synthesis is an evolutionarily conserved process crucial for a wide range of biological processes dependent on a capacious array of enzymes, precursor sugar molecules, and subcellular organelles.^1^ Congenital disorders of glycosylation (CDGs) are an extensive group (>200 disorders) of clinically and genetically heterogeneous conditions arising mainly due to defects in glycoconjugate synthesis and processing.^2^^,^^3^ CDGs are clinically typified by a multisystemic phenotype, frequently involving neurodevelopmental delay, failure to thrive, hepatopathy, and/or coagulopathy.^4^^,^^5^ CDGs are individually rare, classified according to the underlying molecular cause, and are primarily autosomal recessive traits reflecting the catabolic or anabolic nature of most disease-associated genes.^4^ Almost all CDG types present with multisystem disease in infancy, evidencing the fundamental importance of glycosylation for developmental processes (web resources [Congenital disorders of N-linked glycosylation and multiple pathway overview, GeneReviews]). However, a small proportion of CDGs may display more organ-specific or later-onset presentations, including CDG type 1A (MIM: 212065), associated with defects in phosphomannomutase 2 (PMM2), which is characterized by a wide-ranging age of onset and clinical severity of neurological and visceral phenotypes, dependent on the impact of the gene and allelic variant(s) on PMM2 function^6^^,^^7^ (web resources [PMM2-CDG GeneReviews]).

**Figure 1:**
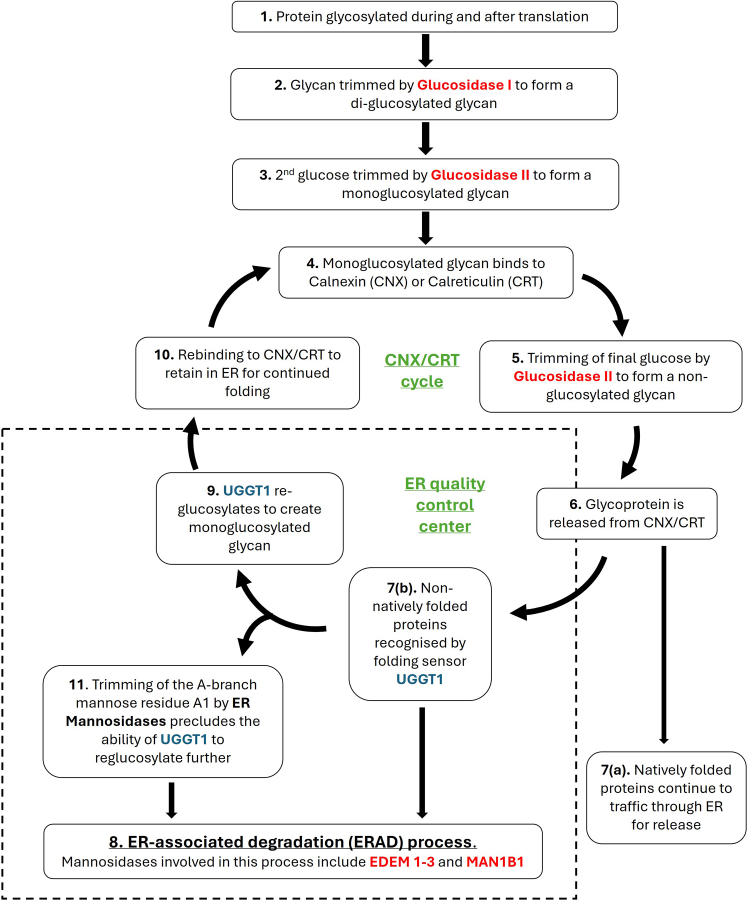
Carbohydrate-dependent quality control pathway Bold text indicates proteins with relevant enzymatic activity. Proteins highlighted in red indicate association with a congenital disorder of glycosylation (CDG) in OMIM. UGGT1 protein is highlighted in blue. Pathway is as follows: (1) protein is glycosylated during and after translation. (2) The resulting glycan attached to the glycoprotein is trimmed by glucosidase I encoded by *MOGS* (MIM: 601336) to form a di-glucosylated glycan. (3) The second glucose is trimmed by glucosidase II (alpha subunit encoded by *GANAB* [MIM: 104160] and beta subunit encoded by *PRKCSH* [MIM: 177060]) to form a monoglucosylated glycan. (4) The monoglucosylated glycan binds to calnexin (CNX), a membrane-bound lectin chaperone, or calreticulin (CRT), CNX’s soluble paralog. This promotes proper folding by preventing aggregation and premature export from the ER. (5) The final glucose is trimmed by glucosidase II to form a non-glucosylated glycan. (6) The resulting glycoprotein is released from CNX/CRT cycle. (7a) Natively folded proteins continue to traffic through the ER for release. (7b) Non-natively folded proteins with minor folding defects are recognized by the folding sensor UGGT1. (8) Terminally misfolded glycoproteins are extracted from the CNX/CRT cycle for degradation by the ER-associated degradation (ERAD) process. This starts by trimming B- and C-branch mannoses to create a degradation signal. These mannosidases include ER mannosidase 1 encoded by *MAN1B1* (MIM: 604346) and EDEM 3 encoded by *EDEM3* (MIM: 610214). (9) UGGT1 reglucosylates the protein to create a monoglucosylated glycan. (10) The glycan re-binds to CNX/CRT to be retained in the ER. The cycle continues from step 5. (11) Alongside this cycle, ER mannosidases trim an A-branch mannose from the glycan, precluding the ability of UGGT1 to reglucosylate, preventing slow folding glycoproteins from remaining in the cycle indefinitely. Once the A1 mannose has been trimmed, the glycoprotein is removed from the cycle for degradation by the ERAD process.

Defining the aberrant glycosylation processes underlying CDGs has driven significant advances in the field of glycobiology, providing invaluable insight into glycoconjugate pathomechanisms.^4^^,^^5^ The most common CDGs arise due to errors in N-linked glycosylation, which are associated with defects in molecules involved in different stages of this biological pathway.^2^ Pathogenic variants in *MOGS* (MIM: 601336), encoding mannosyl-oligosaccharide glycosidase (also known as glucosidase I), disrupt glycan-mediated binding between glycoproteins and the lectin endoplasmic reticulum (ER) chaperones calnexin (CNX) and calreticulin (CRT).^8^ This process is key to achieving native protein folding that facilitates optimal protein localization and function for approximately one-third of the proteome.^5^ CDG type IIb (MIM: 606056; henceforth referred to as MOGS-CDG) is just one of several CDGs caused by failure or disruption of the N-linked glycosylation quality control pathway; others include polycystic kidney disease 3 (GANAB-CDG, MIM: 600666), Rafiq syndrome (MAN1B1-CDG, MIM: 614202), and CDG type IIv (ER degradation-enhancing alpha-mannosidase [EDEM3]-CDG, MIM: 619493).^9^^,^^10^^,^^11^ UDP-glucose:glycoprotein glucosyltransferase 1 (UGGT1) is another fundamental molecule for correct glycoprotein folding via the N-glycoprotein quality control pathway. UGGT1 identifies and reglucosylates misfolded proteins, resulting in ER retention for re-binding to CNX/CRT to enable correct folding.^12^ The process by which UGGT1 binds preferentially to proteins with folding defects is poorly understood. One possible mechanism is that UGGT1 recognizes exposed hydrophobic structures on abnormally folded proteins.^13^ A molecular marking system involving multiple ER-resident exo-mannosidases, including ER mannosidase I (ERManI) and EDEM family members, operates in tandem with this cyclical process by progressively trimming mannose residues from glycoproteins. This stepwise de-mannosylation eventually reduces the affinity of UGGT1 for its substrate, preventing further reglucosylation and facilitating the extraction of misfolded proteins from the CNX cycle (Figure 1).^14^ Disruptions of this pathway lead to an accumulation of misfolded proteins within the ER lumen, which causes ER stress, activating downstream signaling networks that, if unable to restore ER homeostasis, may trigger apoptotic pathways.^5^

UGGT1 has a homolog, UGGT2, which is 55% identical.^12^ Though both isoforms are enzymatically active, UGGT1 is thought to be the primary enzyme responsible for the reglucosylation of misfolded glycoproteins, as exemplified by its expression, which is 26× greater than that of UGGT2 in HeLa cells.^15^ Furthermore, UGGT2 shows a binding preference toward smaller, soluble lysosomal proteins destined for endocytic/lysosomal compartments, as opposed to UGGT1, which favors larger, single-pass proteins destined for secretion and the plasma membrane.^16^ Taking these results together, it is likely there are insufficient levels of UGGT2 to cope with the loss of UGGT1 in addition to substrate specificity issues between the two paralogs.

The fundamental importance of UGGT1 function at the organismal level is evidenced by *Uggt1* knockout mice, which exhibit embryonic lethality at embryonic day (E)13, with only a subset surviving until birth^17^ (web resources [Mouse Genome Informatics UGGT1 mouse model]). Here, we describe our clinical, genetic, and molecular studies of a distinctive but clinically variable CDG due to bi-allelic pathogenic *UGGT1* variants.

## Subjects, material, and methods

### Clinical and genetic studies

**Figure 2:**
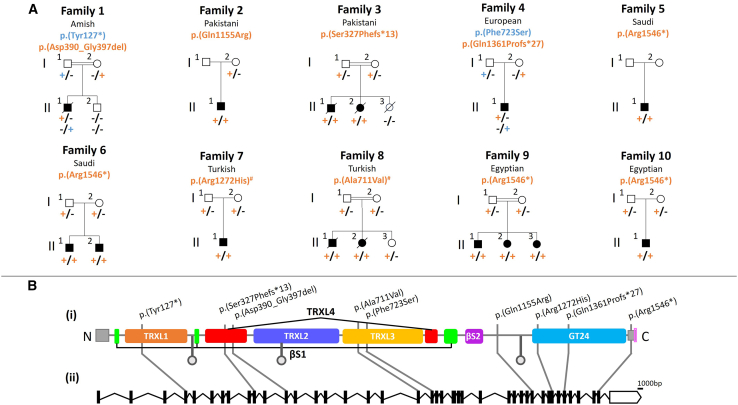
Family pedigrees and bi-allelic *UGGT1* variants associated with UGGT1-CDG (A) Pedigrees of the families investigated depicting autosomal recessive segregation of the pathogenic *UGGT1* variants identified. Co-segregation was confirmed in other family members as indicated; in each case, the plus symbol indicates the variant allele, and the minus sign indicates the wild-type allele. Blue/orange text has been used to differentiate between variants in a pedigree when the UGGT1 genotype in affected individuals is compound heterozygous. # indicates variants that have been shown to impact splicing. (B) (i) Simplified schematic depicting UGGT1 protein structure showing location of each *UGGT1* variant in relation to the predicted domain architecture. Pink shaded box indicates the REEL endoplasmic reticulum retrieval sequence, gray shaded boxes indicate predicted nonsense-mediated decay escape regions, gray circles indicate glycosylation sites, and colored boxes represent protein domains. TRXL1, thioredoxin-like domain 1 (orange); TRXL2, thioredoxin-like domain 2 (purple); TRXL3, thioredoxin-like domain 3 (yellow); TRXL4, thioredoxin-like domain 4 (red); βS1, beta sheet-1 (green); βS2, beta sheet-2 (purple); GT24, glycotransferase 24 (blue). (ii) Schematic of the *UGGT1* MANE select transcript (GenBank: NM_020120.4) depicting intron-exon organization and the location of each *UGGT1* variant.

This study adhered to the principles of the Declaration of Helsinki and was approved by the recruiting institutional review boards (IRBs), with all research participants or their legal guardians providing written informed consent for study participation and the publication of clinical details and photographs. Affected individuals were identified through GeneMatcher, the 100,000 Genomes Project, our collaborative research network, and their clinicians. The families included in this study originated from the following genetic ancestries: Amish (family 1), Pakistani (families 2 and 3), European (family 4), Saudi Arabian (families 5 and 6), Turkish (families 7 and 8), and Egyptian (families 9 and 10) (Figure 2). The affected individuals and their families were identified as a part of larger research or diagnostic programs, and variant interpretation was supported by population-specific allele frequency data, including from the Anabaptist Variant Server (web resources [Anabaptist Variant Server]) for family 1 and the King Faisal Specialist Hospital and Research Centre in Saudi Arabia for families 5 and 6. Pedigrees and detailed phenotypic data for each person were gathered from collaborating clinicians using a standardized clinical proforma. Brain magnetic resonance imaging (MRI) scans (seven cases where data were available) were retrospectively reviewed by author L.M.

DNA was extracted from blood/buccal samples using standard techniques. To identify the cause of the disease, genome sequencing (GS) was performed for families 1–3, while exome sequencing (ES) was done for families 4–10 and S11 (Figure 2). Unless specified, genomic variants were filtered based on call quality, predicted consequence, segregation with disease phenotype, and allele frequency in population databases (variants with a frequency of >0.1% and/or present in >1 homozygous individual in gnomAD v.2.1.1, v.3.1.2, or v.4.1.0 or in-house population-specific databases were excluded). Exonic or intron/exon boundary (±6 nucleotides of the splice junction), homozygous, compound heterozygous, hemizygous, and *de novo* (when trio sequencing performed) variants that remained after filtering were assessed for clinical correlation with the affected individual’s phenotype as previously described at the University of Exeter (family 1, II-1, in Figure 2, Illumina, research GS)^18^; through the 100,000 Genomes Project (family 2, I-1 and II-1, Illumina, research duo GS, and family 3, I-1, I-2, and II-1, Illumina, research trio GS, followed by gene-agnostic filtering)^19^^,^^20^; at GeneDx (family 4, Illumina, diagnostic trio ES)^21^; at the Department of Genetics, King Faisal Specialist Hospital and Research Centre (family 5, II.1, and family 6, II-1, Illumina, research proband only ES)^22^; at the Baylor College of Medicine Human Genome Sequencing Centre (BCM-HGSC) as part of the Baylor-Hopkins Centre for Mendelian Genomics (BHCMG) and BCM-GREGoR (Genomics Research Elucidates the Genetics of Rare Diseases) research program (family 7, II-1, research trio ES; family 8, II-2, research proband only ES; and family 9, II-1, II-2, and II-3, research pentad ES)^23^; at the National Research Centre (NRC) in Cairo, Egypt (family 10, II-1, research proband only ES)^24^; and at University College London (family S11, II:1 and II:2, research duo ES).^25^ In families 1, 3, 4, 6, 7, 8, 9, 10, and S11, dideoxy sequencing co-segregation analysis of *UGGT1* (GenBank: NM_020120.4) (hg38) variants was performed using standard techniques.

### UGGT1 cellular reglucosylation assay

HEK293-6E, *ALG6*^−/−^ (MIM: 604566), and *ALG6*/*UGGT1/2*^−/−^ cells were cultured in DMEM (Sigma D5796), supplemented with 10% fetal bovine serum (Gibco 11965118), and cultured at 37°C and 5% CO_2_. All cell lines were tested for mycoplasma using a universal detection kit (ATCC, cat. #30-012K). Generation of the *ALG6*^−/−^ and *ALG6*/*UGGT1/2*^−/−^ cell lines has been described elsewhere.^26^ For transfection, 1.75E6 cells were added to a 6-cm dish before being returned to the incubator for 24 h. The next day, each plate was transfected with a plasmid encoding wild-type UGGT1, a mutant UGGT1 form containing a C-terminal 3xFLAG tag, or the Z mutant of alpha-antitrypsin 1 (AAT-Z). Briefly, 4.8 μg of DNA was mixed and diluted in Opti-Mem (Gibco 31985070) for a total volume of 80 μL. For the co-transfection of UGGT1^FLAG^ with AAT-Z, the DNA mixture was composed of 50% UGGT1^FLAG^ (2.4 μg) and 50% AAT-Z (2.4 μg). In a separate tube, 12 μL of polyethylenimine (PEI; Polyscience 24765) was added to 68 μL of Opti-Mem and incubated for 5 min at 23°C. The PEI solution was then added to the diluted DNA mixture and incubated at 20 min at 23°C. The entire DNA:PEI solution was then added dropwise to each plate before the cells were placed back in the incubator for an additional 24 h at 37°C and 5% CO_2_.

A detailed protocol of the reglucosylation assay has been described previously.^16^^,^^26^ Briefly, *ALG6*^−/−^ or *ALG6*/*UGGT1/2*^−/−^ cells were transfected with wild-type UGGT1^FLAG^, with a mutant UGGT1 variant, or in combination with AAT-Z. For the activity assay, *ALG6*/*UGGT1/2*^−/−^ cells were used, as no endogenous UGGT1 or UGGT2 was present.

The cells were treated, and the lysate was prepared as described above. The lysate was then divided three ways: 20% (150 μL) for the whole-cell lysate (WCL), 35% (262.5 μL) added to beads precoated with recombinant glutathione S-transferase (GST)-CRT (CRT), and 35% (262.5 μL) added to beads precoated with a lectin dead variant of CRT, GST-CRT (p.Tyr109Ala) (CRT^∗^). The WCL samples were then mixed with 750 μL of cold acetone before incubating overnight at −20°C. The CRT and CRT^∗^ samples were incubated at 4°C using an end-over-end spinner. The next day, the WCL acetone samples were centrifuged at 20,817 × *g*, 4°C, for 10 min before the supernatant was removed, and the pellets were dried for at least 1 h at room temperature. The CRT and CRT^∗^ pull-down samples were spun at 250 × *g* for 3 min, 4°C, to pellet the resin. The supernatant was removed, and the samples were washed three times with 500 μL of cell lysis buffer without protease inhibitor. After the final spin, 35 μL of gel loading buffer was added to all tubes, and the samples were treated as described above before being resolved by 9% SDS-PAGE gel and transferred to a PVDF membrane. The membrane was probed with either a 3xFLAG mouse monoclonal antibody to detect UGGT1^FLAG^ and AAT-Z or a UGGT1 rabbit polyclonal antibody (GeneTex GTX66459) to detect endogenous UGGT1 or UGGT1^FLAG^. The membranes were then incubated with either a rabbit secondary antibody (LI-COR 926–68071) to detect UGGT1 or a mouse secondary antibody to detect 3xFLAG.

To determine the percentages of glucosylation and activity, the protein levels in the WCL, CRT, and CRT^∗^ lanes were quantified using ImageJ. The levels obtained were then normalized by their corresponding input percentages. The level of glucosylation was then calculated by first subtracting the background in the CRT^∗^ lane from the amount quantified in the CRT lane before dividing the adjusted value by the amount in the WCL. The resulting value was then multiplied by 100 to obtain the percentage of glucosylation. All blots are representative of three independent biological replicates. The standard deviation is displayed for all activity and glucosylation quantifications. Statistical significance was determined by using an unpaired t test with a confidence interval of 95%.

### Catalytic activity assay

This enzymatic assay was undertaken as previously described.^12^^,^^27^ To ensure that the concentrations of the UGGT1 mutants were consistent throughout all assays, bead suspensions that yielded the same level of Coomassie brilliant blue (CBB) staining on SDS-PAGE were used. Gel images were captured using ImageQuant LAS 4000 (GE Healthcare, Buckinghamshire, UK) and processed using ImageJ software, followed by an adjustment of the protein concentration of each sample with suspension buffer. Briefly, a reaction mixture containing 80 nM of the acceptor substrates M9-Asn-BODIPY and M9-Gly-BODIPY, 2 mM UDP-Glc, 2 μL UGGT on M2 agarose beads, 2 mM CaCl2, 0.05% Triton X-100, and 10 mM HEPES (pH 7.4) was prepared in a total volume of 20 μL and the solution incubated at 37°C for 3 h, followed by reaction quenching using 10 μL of acetonitrile. The percentage of glucose transfer in each reaction was quantified by high-performance liquid chromatography (HPLC) under the following conditions: InertSustain Amide column (3 μm, 4.6 × 150 mm), mobile phase CH3CN/10 mM ammonium formate (pH 4.5), gradient from 85:15 to 41:59 in 45 min, and a flow rate of 0.75 mL/min at 40°C. BODIPY-labeled glycans were quantified based on the fluorescence intensity (λex = 488 nm, λem = 520 nm) using a Waters 2475 fluorescence detector.

### Cell culture

Skin fibroblasts from healthy control subjects GM-00038, GM-05567, GM-09503, GM-01652, GM-05381, and GM-08447 (Coriell) and affected individuals II:1 (BAB15130) and II:2 (BAB15130) from family 9 were cultured in 1 g/L glucose DMEM (Corning) supplemented with 10% heat-inactivated FBS (Sigma), 1× L-glutamine (Corning), and 1× penicillin-streptomycin (Corning).

### UGGT1 localization studies

The day after the cells were transfected with the specified C-terminal UGGT1^FLAG^ construct, the media and cells were collected. The media were spun gently at 250 × *g* for 5 min at 23°C to remove any cellular debris. Once cleared, 20 μL of Protein-A-Sepharose 4B resin (Invitrogen, 101042) and 1 μL of a 3xFLAG mouse monoclonal antibody (Sigma F1804) were added to each sample to immunoprecipitate secreted UGGT1^FLAG^. The tubes were then incubated overnight at 4°C using an end-over-end rotation. To prepare the WCL fraction, the cells were first washed with 1 mL of PBS. The cells were then placed on ice, and 750 μL of lysis buffer (20 mM MES, 100 mM NaCl, 30 mM Tris [pH 7.5], and 0.5% Triton X-100) containing a protease inhibitor cocktail (Thermo 1861278) and 20 mM N-ethylmaleimide (NEM) was added to each plate. The cells were then scraped off the plate, and the lysate was shaken vigorously for 10 min at 4°C before centrifuging for 10 min at 20,817 × *g*, 4°C. 20% of the lysate (150 μL) was then mixed with 750 μL of ice-cold acetone and incubated overnight at −20°C to precipitate cellular proteins.

The next morning, the acetone samples were centrifuged at 20,817 × *g*, 4°C, for 10 min. The supernatant was removed, and the pellets were dried at room temperature for at least 1 h. The media immunoprecipitation samples were spun gently at 500 × *g* for 3 min at 23°C. The supernatant was removed, and 500 μL of PBS was added to each tube before being spun again for 3 min at 500 × *g*, 23°C. The beads were washed twice more. 35 μL of gel loading buffer (30 mM Tris-HCl [pH 6.8], 9% SDS, 15% glycerol, and 0.05% bromophenol blue) containing 100 mM dithiothreitol (Sigma D9779) was added to all samples before they were treated for 10 min at 95°C and shaken vigorously. 15 μL of each sample was then resolved on a 4%–20% gradient gel (Bio-Rad 4561096) before being transferred to a PVDF membrane (Millipore IPFL00010). The membranes were then probed with a 3xFLAG mouse monoclonal antibody (1:5,000) before using a mouse secondary (LI-CORE 926–32210) for fluorescence detection.

The percentage secreted was calculated by first quantifying the amount of UGGT1 in the lysate and the corresponding media lanes using ImageJ. The quantities were then added together to obtain the total amount of UGGT1 produced. The quantity in the media was then divided by the total amount of protein and multiplied by 100 to obtain the percentage of UGGT1 in the media. All blots are representative of three separate biological replicates. The standard deviation is displayed for all quantifications of UGGT1 secretion. Statistical significance was determined by using an unpaired t test with a confidence interval of 95%.

For the localization study using affected individuals’ skin fibroblasts, cultured fibroblasts were grown to ∼70% confluence and collected using a cell scraper, spun down at 1,600 rpm for 4 min, and subsequently washed thrice with DPBS. Cell pellets were lysed in 2% SDS lysis buffer containing 62.5 mM Tris (pH 6.8) and 10% glycerol and sonicated for 10 s to completely dissociate the cell pellet. The resulting WCL was then boiled at 100°C for 10 min. Protein concentrations were determined using a Nanodrop (Thermo Fisher Scientific). WCLs (∼30 μg) were resolved on a 6% SDS-PAGE gel. Immunoblotting was performed using a polyclonal anti-UGGT1 (Bethyl Labs A305-529A) at a dilution of 1:1,000. For blots containing the secreted UGGT1 in the culture medium, the same exact protocol was performed with the following modifications. Fibroblasts were split into and cultured in a 6-cm plate. The following day, the culture medium was changed to a reduced volume of 2 mL, and the fibroblasts were allowed to grow for 3 days without media changes or additions. Cell pellets were collected as above, and the medium was collected and filtered through a microfilter to remove cell debris. A total of 24 μL undiluted medium was run on the same 6% SDS-PAGE gel as the WCL (Corning). Rabbit polyclonal anti-UGGT1 was used for western blot studies (Bethyl Labs A305-529A) (1:1,000).

### Mini-gene splicing assay

A mini-gene splicing assay was performed using a mini-gene split GFP construct,^28^ in which the N- and C-terminal parts of the GFP gene were separated by *SMN1* (MIM: 600354) introns 7 and 8 (GenBank: NM_000344). Reference and mutated gene fragments containing 400–500 bp including and surrounding exons 19, 22, or 34 of the *UGGT1* gene flanked with 30-bp vector homology arms were synthetized (TWIST Bioscience, USA) and cloned into the mini-gene construct (Gibson Assembly Master Mix, New England Biolabs). After Sanger sequencing verification of all constructs, they were transfected into the HEK293 cells (Lipofectamine 3000, Thermo Fisher Scientific). 48 h post-transfection, total RNA was extracted from the transfected cells (RNAeasy Mini Kit, Qiagen), and cDNA was generated using random hexamer primers (SuperScript IV Synthesis Kit, Thermo Fisher Scientific). Subsequently, the mini-gene transcripts were amplified from the cDNA using primers specific to the split GFP fragments (forward [F]: 5′-CACACTGGTGACAACATTTACATAC-3′; reverse [R]: 5′-GAAATCGTGCTGTTTCATGTGATC-3′). The PCR products were column purified (DNA Clean & Concentrator-5, Zymo Research) and analyzed with next-generation amplicon sequencing (MiSeq, Illumina, OGI Genomics Core). The splicing pattern analysis was performed by aligning the sequence reads to the hybrid reference of the split GFP construct containing the DBT intron 10 (STAR Aligner)^29^ and visualizing the reads in the Integrated Genome Viewer (IGV).

## Results

### Genetic analysis

We initially investigated the cause of disease in a male Amish infant (family 1, II:1) (Figure 2A) presenting shortly after birth with central apneas, microcephaly, seizures, central hypotonia, limb hypertonia, craniofacial dysmorphism with coarse features, clenched hands with overlapping fingers and toes, cryptorchidism, thoracic scoliosis, hepatomegaly, and enlarged cystic kidneys, raising suspicion of autosomal recessive polycystic kidney disease. He died at 5 months of age due to respiratory failure. Pertinent investigations included electroencephalography (EEG) indicative of active epilepsy overlying a significant degree of encephalopathy and cranial MRI (2 weeks of age) showing periventricular heterotopia lining the bodies of the lateral ventricles. An abdominal ultrasound scan revealed bilaterally enlarged kidneys with markedly abnormal increased cortical echogenicity with small cystic foci alongside hepatomegaly with diffuse increased periportal echogenicity and common and intrahepatic bile duct dilatation.

To define the genetic cause of the disease, GS was performed on DNA from the affected child (family 1, II-1). Filtering of GS data using standard metrics described above identified standout candidate compound heterozygous variants in *UGGT1* (GenBank: NM_020120.4): g.128108041_128108044del (c.381_384del [p.Tyr127^∗^]) and g.128127394_128127417del (c.1168_1191del [p.Asp390_Gly397del]). The c.381_384del (p.Tyr127^∗^) variant is located in exon 4/41, is present at low frequency in gnomAD (0.0000006), with no homozygotes recorded, and is predicted to result in premature termination and nonsense-mediated decay (NMD). The c.1168_1191del (p.Asp390_Gly397del) variant is absent in gnomAD (gnomAD v.2.1.1, v.3.1.2, and v.4.1.0, accessed on November 18, 2024) (Table 2) and is predicted to cause an in-frame deletion within the thioredoxin-like domain 4 (TRXL4) required for substrate binding, most likely resulting in loss of catalytic activity of the resulting protein (Figure 2B). The variants co-segregate appropriately in all family members (Figure 2A) and, as expected for Amish founder variants, are also present in the Anabaptist (Amish and Mennonite) variant server^30^ (a population-specific database comprising >10,000 exomes) at low-allele frequencies (0.0003 and 0.0001, respectively) and only in the heterozygous state.

To further investigate the hypothesis that bi-allelic deleterious variations in *UGGT1* underlie human developmental disorder traits, we clinically characterized 15 affected individuals from 10 unrelated families identified through GeneMatcher and international rare disease research collaborations, harboring putative pathogenic bi-allelic *UGGT1* variants (Table 1).^31^

**Table 1:** Clinical findings in individuals with UGGT1-CDG

**Family**	**Sex, ethnicity**	**Age at last exam**	**Genotype (GeneBank:**NM_020140.4**)**	**Craniofacial dysmorphism**	**OFC birth (*Z* score)**	**OFC (*Z* score)**	**Developmental delay**	**Intellectual disability/IQ testing**	**Muscle tone**	**Seizures**	**Autistic features**	**Brain MRI**	**Genitourinary anomalies**	**Hepatobiliary anomalies**	**Cardiac anomalies**	**Skeletal anomalies**	**Ocular anomalies**	**Other features**	**Transferrin testing**
**Family 1**																			

Individual II:1	male, Amish	deceased, 5 months	c.381_384del (p.Tyr127^∗^), heterozygous; c.1168_1191del (p.Asp390_Gly397del), heterozygous	+	34.5 cm (−1.02)	38.1 cm; 4 months (−3.95)	severe	N/A	central hypotonia, limb hypertonia	neonatal onset tonic-clonic	N/A	periventricular heterotopia, mild delay in myelination	enlarged kidneys with cystic foci, cryptorchidism	hepatomegaly with periportal echogenicity/fibrosis	PFO, PDA	Overlapping fingers and toes, scoliosis, wide sandal gap	horizontal nystagmus	central apnea, feeding difficulties, hearing impairment, respiratory failure	normal

**Family 2**																			

Individual II:1	male, Pakistani	17 years, 7 months	c.3464A>G (p.Gln1155Arg), homozygous	−	33.5 cm (−1.14)	52.8 cm; 9 years, 10 months (−1.02)	severe and nonverbal	severe	normal	severe, medically refractory	autism, challenging behavior	normal	−	−	−	−	−	−	normal

**Family 3**																			

Individual II:1	male, Pakistani	deceased, 7 months	c.978_979del (p.Ser327Phefs^∗^13), homozygous	+	32.5 cm (−0.66)	NK	severe	N/A	limb contractures	−	N/A	periventricular heterotopia, hypoplastic cerebellum and optic nerves	cystic renal dysplasia, cryptorchidism, short penis	intra- and extrahepatic bile duct dilation	hypoplastic aortic arch, PDA, ASD	contractures, upper thoracic hemivertebra	bilateral coloboma	CLP, SN hearing loss, FTT, hernias, respiratory failure, aortic and IVC thrombi	N/D
Individual II:2	female, Pakistani	deceased, fetus	c.978_979del (p.Ser327Phefs^∗^13), homozygous	N/A	N/A	N/A	N/A	N/A	N/A	N/A	N/A	cystic post-fossa (antenatal scan)	NK	NK	DORV, TGA, VSD, hypoplastic pulmonary valve with PS	NK	N/A	CLP, cystic abdominal mass, lung hypoplasia	N/A

**Family 4**																			

Individual II:1	male, European	20 yo	c.2168T>C (p.Phe723Ser), heterozygous; c.4081dupC (p.Gln1361Profs^∗^27), heterozygous	+	NK	54.6 cm; 16 years, 4 months (−1.25)	severe and nonverbal	severe	hypotonia (infancy), normal	febrile onset then afebrile, partial complex, and GTC	autism, severe self-injury	NK	−	−	−	−	NK	hypothyroidism	N/D

**Family 5**																			

Individual II:1	male, Saudi	14 yo	c.4636C>T (p.Arg1546^∗^), homozygous	+	NK	NK	+	NK	lower limb hypertonia	recurrent febrile	hyperactive	normal	NK	NK	NK	NK	NK	−	N/D

**Family 6**																			

Individual II:1	male, Saudi	9 yo	c.4636C>T (p.Arg1546^∗^), homozygous	+	NK	microcephaly	severe	severe	hypotonia (infancy), hypertonia	focal	hyperactive	normal	−	−	−	−	−	−	N/D
Individual II:2	male, Saudi	14 yo	c.4636C>T (p.Arg1546^∗^), homozygous	+	NK	51.5 cm (−2.63)	severe	severe	NK	febrile	hyperactive, stereotypies	normal	cryptorchidism	NK	−	NK	NK	−	N/D
**Family 7**																			

Individual II:1	male, Turkish	10 yo	c.3815G>A (p.Arg1272His), homozygous	+	37 cm (1.41)	51 cm (−2.18)	severe	severe	normal	absence, myoclonic, and GTC	stereotypies, anxiety	bilateral high T2/low T1 signal in the putamina	−	−	−	−	strabismus	−	N/D

**Family 8**																			

Individual II:1	male, Turkish	deceased, 8 yo	c.2132C>T (p.Ala711Val), homozygous	NK	33.7 cm (−1.18)	NK	severe	severe	NK	febrile clonic seizures	NK	hypoplastic CC	bilateral dilated pyelum and hydronephrosis, mega-ureter	NK	bicuspid aortic valve, PDA	NK	NK	cyclic neutropenia	N/D
Individual II:2	female, Turkish	deceased, 7 yo	c.2132C>T (p.Ala711Val), homozygous	+	32.5 cm (−0.95)	45.5 cm, 3 yo (−4.01)	severe and nonverbal	severe	central hypotonia	febrile clonic seizures	stereotypies	L mesial temporal sclerosis, asymmetric cerebral hemisphere atrophy	−	−	small VSD	−	cortical blindness	cyclic neutropenia	normal

**Family 9**																			

Individual II:1	female, Egyptian	10 years, 7 months	c.4636C>T (p.Arg1546^∗^), homozygous	+	33 cm (−1.20)	46.5 cm (−5.85)	severe and nonverbal	severe, IQ 25	hypertonia	tonic, GTCs	autism, hyperactive	hypoplastic CC	−	−	−	arachnodactyly	−	hirsutism	N/D
Individual II:2	female, Egyptian	8 years, 1 month	c.4636C>T (p.Arg1546^∗^), homozygous	+	33.5 cm (−0.90)	48.5 cm (−3.43)	severe	severe, IQ 35	hypertonia	−	autism, hyperactive	bright hippocampi	−	−	−	arachnodactyly	−	hirsutism	N/D
Individual II:3	male, Egyptian	6 years, 3 months	c.4636C>T (p.Arg1546^∗^), homozygous	+	34 cm (−0.95)	49 cm (−2.77)	severe and nonverbal	severe, IQ 30	hypertonia	tonic seizures	autism, hyperactive	abnormal T2 signal in anterior temporal lobes	−	−	−	arachnodactyly	−	hirsutism	N/D

**Family 10**																			

Individual II:1	male, Egyptian	11 months	c.4636C>T (p.Arg1546^∗^), homozygous	+	34 cm (−0.47)	42.5 cm (−3.97)	moderate	N/A	hypertonia	−	autism, anxiety	thin CC	−	−	−	−	nystagmus	−	N/D
Summary	N/A	N/A	N/A	12/13 (93%)	N/A	9/11 (81%)	14/14 (100%)	10/10 (100%)	N/A	11/14 (73%)	11/11 (100%)	10/14 (71%)	4/13 (31%)	2/11 (18%)	5/14 (36%)	5/11 (45%)	5/10 (50%)	N/A	N/A

+, presence of a feature; −, absence of a feature; NK, not known; N/A, not applicable; N/D, not done; L, left; R, right; yo, years old; mo, months; ASD, atrial septal defect; CC, corpus callosum; CLP, cleft lip and palate; DORV, double outlet right ventricle; FTT, failure to thrive; GTC, generalized tonic clonic; IVC, inferior vena cana; OFC, occipitofrontal circumference; PDA, patent ductus arteriosus; PFO, patent foramen ovale; PS, pulmonary stenosis; SN, sensorineural (hearing loss); TGA, transposition of the great arteries; VSD, ventricular septal defect.

ES/GS studies in each family identified nine *UGGT1* variants as the likely cause of disease, including one nonsense variant (c.4636C>T [p.Arg1546^∗^]), four insertion or deletion (indel) variants (c.381_384del [p.Tyr127^∗^], c.978_979del [p.Ser327Phefs^∗^13], c.1168_1191del [p.Asp390_Gly397del], and c.4081dupC [p.Gln1361Profs^∗^27]), and four missense variants (c.2132C>T [p.Ala711Val], c.2168T>C [p.Phe723Ser], c.3464A>G [p.Gln1155Arg], and c.3815G>A [p.Arg1272His]). All variants are ultra-rare or absent from gnomAD v.4.1.0. All missense variants are highly conserved from humans to zebrafish, with less stringent conservation in yeast. The only exception is the p.Ala711Val variant, which is conserved in all mammals (Figure S1). Missense variants are predicted to be deleterious by CADD/REVEL; furthermore, there are no homozygous loss-of-function variants in the canonical *UGGT1* transcript listed in gnomAD v.4.1.0 (Figure 2B; Table 2). The indels p.Tyr127^∗^ and p.Ser327Phefs^∗^13 are predicted to undergo NMD and, therefore, likely represent null alleles. Two of the four missense variants arise from variants impacting bases adjacent to intron-exon splice junctions. p.Ala711Val alters the second-to-last amino acid in exon 19, p.Arg1272His alters the last amino acid of exon 34, and both are predicted to cause *UGGT1* splicing abnormalities by SpliceAI (Table 2). All *UGGT1* variants identified were found to co-segregate in all available family members, consistent with Mendelian expectations (Figure 2A).

**Table 2:** UGGT1 variants identified in this study

**Family**	**Genomic location, Chr2: (GRCh38)**	**c.****NM_020120.4****(MANE select)**	**p. NP_064505.1**	**Zygosity**	**gnomAD v.2.1.1 MAF**	**gnomAD v.4.1.0 MAF**	**No. of homs gnomAD v.4.1.0**	***In silico* predictions**			
								**Revel**	**SpliceAI**	**CADD**	**ACMG classification**
1	g.128108041_ 128108044del	c.381_384del	p.Tyr127^∗^	Het	0	0.0000006196	0	N/A	0.24	N/A	PVS1, PS3_Mod, PM2 (P)
	g.128127394_ 128127417del	c.1168_1191del	p.Asp390_Gly397del	Het	0	0	0	N/A	0.14	N/A	PS3_Mod, PM2, PM3, PM4 (LP)
2	g.128174783A>G	c.3464A>G	p.Gln1155Arg	Hom	0	0.000001240	0	0.654	0	25.9	PS3_M, PM2, PM3_Supp, PP3 (LP)
3	g.128121203_ 128121204del	c.978_979del	p.Ser327Phefs^∗^13	Hom	0	0	0	N/A	0.02	N/A	PVS1, PM2, PM3_Supp, PP1_Mod (P)
4	g.128181070dupC	c.4081dupC	p.Gln1361Profs^∗^27	Het	0	0. 0000006196	0	N/A	0.01	N/A	PVS1, PM2 (LP)
	g.128155519T>C	c.2168T>C	p.Phe723Ser	Het	0	0.000002479	0	0.582	0.01	24.4	PS3_Mod, PM2, PM3 (LP)
5, 6, 9, 10	g.128187608C>T	c.4636C>T	p.Arg1546^∗^	Hom	0.000004015	0.000004343	0	0.026	0	34	PVS1_Str, PS3_Mod, PM2, PP1_Str (P)
7	g.128178569G>A	c.3815G>A	p.Arg1272His	Hom	0.000004028	0.000003111	0	0.519	0.64	36	PVS1_Str (RNA), PM2, PM3_Supp (LP)
8	g.128152899C>T	c.2132C>T	p.Ala711Val	Hom	0	0.000003100	0	0.123	0.92	33	PVS1 (RNA), PM2 (LP)

ACMG, American College of Medical Genetics; Het, heterozygous; Hom, homozygous; MAF, minor-allele frequency; (P), pathogenic; (LP), likely pathogenic.

In addition to these 10 families, we identified a further family with two affected individuals carrying a homozygous *UGGT1* splice site variant (c.2355+4A>G, p.?) (family S11) of uncertain significance (VUS). Both affected individuals are also homozygous for a missense VUS in *FCSK* (MIM: 608675). Bi-allelic *FCSK* variants have been associated with CDG with defective fucosylation (MIM 618324).^32^ The phenotype of the siblings would be consistent with both CDGs; however, it has not been possible to determine the pathogenicity and respective contributions of each variant to the phenotype in this family. Further details are provided in the supplemental information (Figure S2; Table S1).

Interestingly, the p.Arg1546^∗^ variant was identified in four unrelated families from Saudi Arabia (families 5 and 6) and Egypt (families 9 and 10), is located in the penultimate exon, and is predicted to remove ten C-terminal amino acids. Analyses of the whole-exome sequencing (WES) data from all affected individuals carrying this variant show a shared surrounding haplotype, consistent with this being an Arab founder variant.

### Clinical features of *UGGT1* CDG

**Figure 3:**
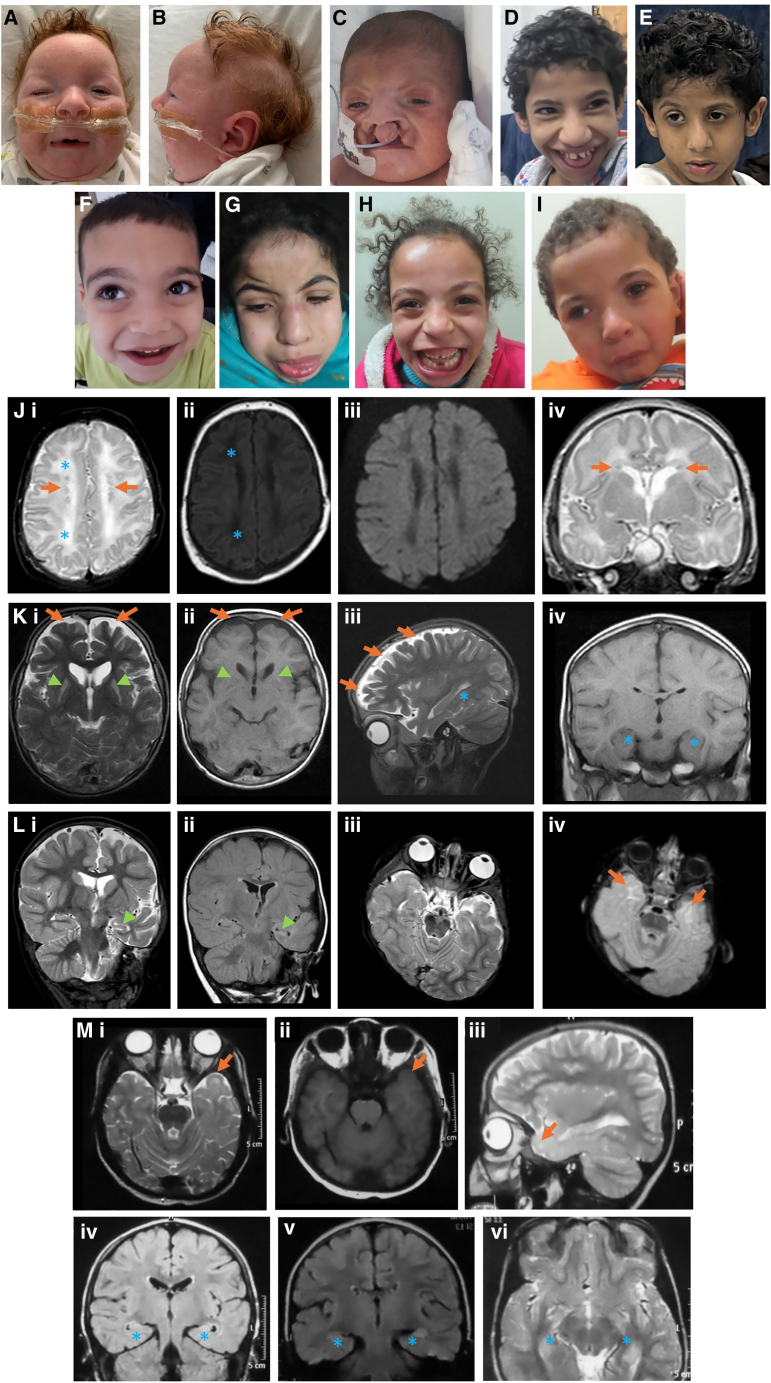
Facial features and neuroimaging findings seen in individuals with UGGT1-CDG (A and B) Individual II-1 from family 1 showing coarse facial features, micrognathia, microcephaly with a sloping forehead, prominent widely spaced eyes/narrow palpebral fissures, broad nasal bridge with upturned bulbous nose, and low set and posteriorly rotated ears. (C) Individual II-1 from family 3 has features that include bilateral cleft lip, high nasal root with broad nasal bridge, narrow palpebral fissures, and arched eyebrows. (D and E) Individuals II-1 (D) and II-2 (E) from family 6 showing microcephaly, bulbous nasal tip, and triangular facies. (F) Individual II-1 from family 7 shows hypertelorism, wide and prominent central incisors, mild bifid nasal tip, and strabismus. (G–I) Individuals II-1 (G), II-2 (H), and II-3 (I) from family 9 showing coarse facies, long face, arched eyebrows, high forehead, broad nasal root, prominent nasal bridge, barrel nose, long smooth philtrum, everted lower lip, macrostomia, protruded tongue, and low set ears. (J) Family 1, II:1. MRI was performed at 2 weeks of age. Axial T2 (i), T1 (ii), and DWI (iii) and coronal T2 (iv) showing mildly abnormal white matter, which may be immaturity or edema in both corona radiata (i and ii, blue asterisk) with no restricted diffusion (iii). Periventricular heterotopic gray matter (i, ii, and iv, orange arrows). (K) Family 7, individual II:1. Axial T2 (i) and T1 (ii), sagittal T2 (iii), and coronal T1 (iv) showing lack of frontal volume (i, ii, and iii, orange arrows). Bilateral high T2/low T1 signal in the putamina bilaterally (i and ii, green arrowheads). Hippocampi appear normal (iii and iv, blue asterisk). (L) Family 8, individual II:2. MRI coronal T2 (i) and FLAIR (ii), axial T2 (iii), and proton density (iv) performed at 3 years of age demonstrating left mesial temporal sclerosis (i and ii, green arrowhead), impaired myelination temporal white matter (iv, orange arrows), and left cerebral hemisphere atrophy (all images). (M) Family 9, individuals II:3 (i–iv) and II:2 (v and vi). II:3: axial T2 (i) and T1 (ii), sagittal T2 (iii), and coronal FLAIR (iv). Abnormal T2 signal in anterior temporal lobes (i and iii, orange arrow), without low T1 signal (ii, orange arrow) suggesting insult. Possible increased T2/FLAIR signal in hippocampi (iv, blue asterisk). II:2: coronal FLAIR (v) and axial T2 (vi). Bilateral increased T2/FLAIR signal in hippocampi (v and vi, blue asterisk) without other associated abnormalities.

Table 1 summarizes the core clinical features of all 15 affected individuals (11 males and 4 females) from 10 unrelated families of diverse ethnic backgrounds. The ages at last examination ranged from 5 months to 20 years. The phenotypic spectrum ranged from fetal demise/infantile death and multiorgan system involvement (families 1 and 3) to a complex syndromic neurodevelopmental disorder (families 2 and 4–10). Individuals who survived the first year of life uniformly had severe global developmental delay (GDD)/intellectual disability. Other findings seen in over half of the cohort include dysmorphic features, microcephaly (defined as ≥ to −2 SDs; *Z* score ranging from −1.02 to −5.85), and seizures (Figure 3). While a recognizable facial gestalt could not be appreciated from clinical descriptions or available photographs, distinctive craniofacial features included micrognathia, long or triangular facies, coarse features, arched eyebrows, sloping forehead, hypertelorism, epicanthal folds, broad or high nasal bridge, upturned bulbous nose, bifid nasal tip, short or smooth philtrum, abnormal teeth, and low set, posteriorly rotated ears (Figures 3A–3I). Skeletal deformities occurred in a subset of patients and included overlapping fingers and toes, scoliosis, abnormal vertebrae, 3–4 finger and toe syndactyly, wide sandal gap, arachnodactyly, and metatarsus varus (Figure S3). Seizure types included febrile, generalized tonic-clonic, tonic, myoclonic, and complex partial; in some cases, these were drug resistant or required polytherapy. Behavioral traits were almost universally present and included features associated with autism spectrum disorder, such as anxiety, stereotyped movements (e.g., hand flapping), self-injurious behaviors, and hyperactivity. Congenital heart disease was seen in five affected individuals and included transposition of the great arteries, aortic coarctation and hypoplastic aortic arch, double outlet right ventricle, atrial septal defect (ASD), ventricular septal defect (VSD), and bicuspid aortic valve and open ductus arteriosus. Other rare associations included genitourinary anomalies (observed in four affected individuals), including most commonly cryptorchidism in males. Cystic renal dysplasia and hepatobiliary anomalies reminiscent of autosomal recessive polycystic kidney disease were reported in two unrelated individuals.

Neuroimaging abnormalities were frequently appreciated (Figure 3; Table 1) but were nonspecific and included gray matter heterotopia, abnormal myelination, corpus callosum thinning, and cerebellar vermian atrophy versus hypoplasia (Figures 3J–3M). Neuroimaging was reportedly normal in two individuals with the homozygous *UGGT1* p.Arg1546^∗^ variant (families 5 and 6). EEG was often abnormal (8/11), including diffuse slowing, severe discontinuity, focal or multifocal sharp waves or spikes, generalized spike/multiple spike slow waves, bilateral slow waves, and electrographic seizures. Clinical carbohydrate-deficient transferrin testing was performed in three affected individuals from families 1, 2, and 8 and reported as unremarkable; no other individuals underwent carbohydrate-deficient transferrin testing.

### *UGGT1* variants disrupt glucosyltransferase activity

**Figure 4:**
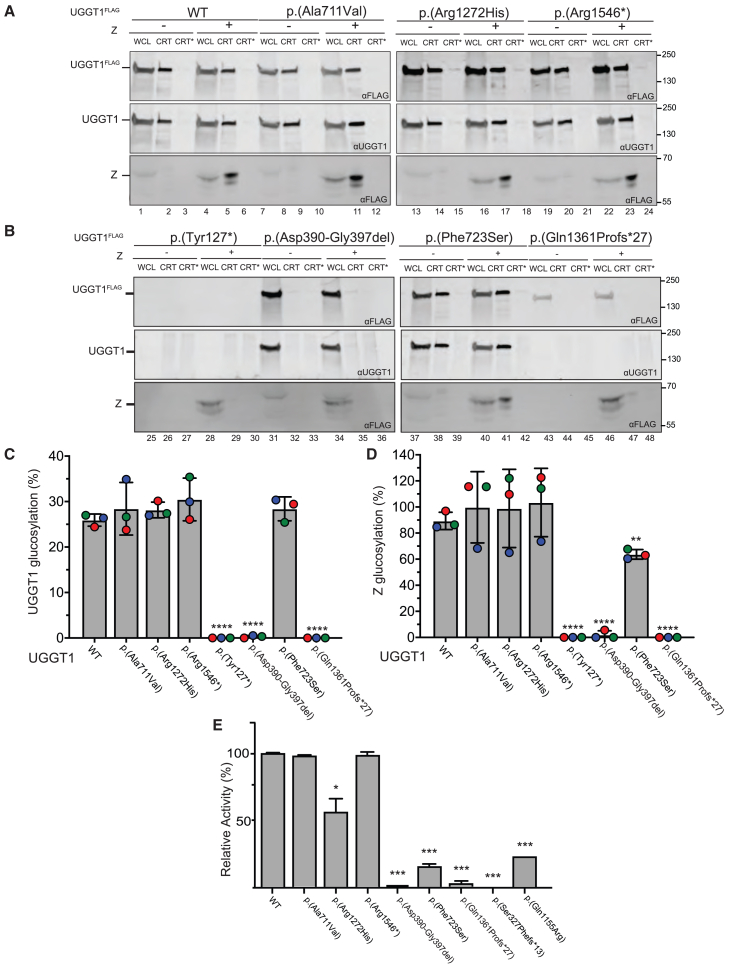
*UGGT1* variants disrupt UGGT1-mediated glucosylation and glucosyltransferase activity (A) Wild-type (WT) UGGT1 (lanes 1–6), p.Ala711Val (lanes 7–12), p.Arg1272His (lanes 13–18), and p.Arg1546^∗^ (lanes 19–24) were expressed individually or co-transfected with AAT-Z in *ALG6*/*UGGT1/2*^−/−^ cells. Once prepared, the cellular lysates were split between whole-cell lysates (WCLs) (20%) to detect total protein, 35% for CRT to isolate monoglucosylated UGGT1 or AAT-Z, and 35% to CRT^∗^ to detect any background binding to the recombinant GST-CRT protein. The samples were resolved by 9% SDS-PAGE gel before being probed by western blot. Immunoblots were probed with an anti-FLAG polyclonal antibody to measure the expression and reglucosylation of exogenous UGGT1 (top) and AAT-Z (bottom). The immunoblots were also probed with an anti-UGGT1 polyclonal antibody to ensure bands were specific to overexpressed UGGT1 (middle). (B) Western blot measuring the activity of WT UGGT1 and heterozygous UGGT1 mutants. UGGT1 p.Tyr127^∗^ (lanes 1–6), p.Asp390-Gly397del (lanes 7–12), p.Phe723Ser (lanes 13–18), and p.Gln1361Profs^∗^27 (lanes 19–24) were expressed along with AAT-Z, prepared, and analyzed as in (C)–(E). The endogenous UGGT1 antibody was raised against a fusion protein containing the amino acid sequence 1,456–1,555 for human UGGT1 and thus is unable to detect mutants missing this sequence. (C and D) Quantification of UGGT1 (C) and AAT-Z (D) glucosylation from (A) and (B). Percentage of reglucosylation was determined by dividing the amount of quantified CRT, subtracting any background from the CRT^∗^, and dividing it by the WCL before multiplying it by 100. Error bars represent standard deviation. ^∗∗^*p* < 0.01 and ^∗∗∗∗^*p* < 0.0001, respectively. Colored circles represent replicates. All western blots are representative of three separate biological replicates. (E) Glucosyltransferase activity of recombinant UGGT1 proteins harboring each variant as a percentage of WT glucosyltransferase activity. p.Tyr127^∗^ variant protein could not be detected by western blot and so has been omitted. Error bars represent standard deviation. ^∗^*p* < 0.05 and ^∗∗∗^*p* < 0.001.

To investigate the biological significance of *UGGT1* variants identified in affected individuals, we employed two complementary approaches: a cell-based glucosylation assay and an *in vitro* catalytic activity assay (Figure 4). The cell-based glucosylation assay utilizes *ALG6*^−/−^ cells, which transfer Man_9_GlcNAc_2_ to glycoproteins rather than the mature Glc_3_Man_9_GlcNAc_2_ glycan (Figure S4).^16^ In the absence of ALG6, lectin chaperone binding is solely dependent on glucosylation by UGGT; glucosylation of the AAT-Z is absent in cells lacking ALG6 and UGGT1/2 (Figures S4B and S4C).^26^ Transfection of wild-type UGGT1 into *ALG6*/*UGGT1/2*^−/−^ cells restores trans-glucosylation of AAT-Z and *self*-glucosylation of UGGT1, whereas transfection of UGGT1 catalytic site mutants reduces or completely abolishes UGGT1 and AAT-Z glucosylation (Figure S5). Thus, *ALG6*/*UGGT1/2*^−/−^ cells provide a useful model to study the activity of *UGGT1* variants.

Using the *ALG6*/*UGGT1/2*^−/−^ cellular model, we examined the impact of seven of the *UGGT1* variants identified in affected individuals (p.Ala711Val, p.Arg1272His, p.Arg1546^∗^, p.Tyr127^∗^, p.Asp390_Gly397del, p.Phe723Ser, and p.Gln1361Profs^∗^27) (Figures 4A–4D). *UGGT1* p.Tyr127^∗^, p.Asp390_Gly397del, and p.Gln1361Profs^∗^27 result in a significant loss of UGGT1 *self*-glucosylation and *trans*-glucosylation of AAT-Z, which was below the limit of detection. *UGGT1* p.Phe723Ser results in significantly reduced *trans*-glucosylation of AAT-Z but did not impact UGGT1 *self*-glucosylation (Figures 4A–4D). The remaining *UGGT1* variants, p.Ala711Val, p.Arg1272His, and p.Arg1546^∗^, did not significantly impact UGGT1 or AAT-Z glucosylation.

We next examined the impact of all identified variants on UGGT1 protein catalytic activity using our *in vitro* enzymatic activity assay. The majority of variants were shown to cause partial to full loss of enzymatic activity of UGGT1: p.Tyr127^∗^, p.Asp390_Gly397del, p.Phe723Ser, p.Gln1155Arg, and p.Gln1361Profs^∗^27 cause highly significant loss of activity (*p* < 0.001, *n* = 3), while p.Arg1272His causes significant loss of activity (*p* < 0.05, *n* = 3). The p.Ala711Val and p.Arg1546^∗^ variants showed no significant reduction in enzyme activity compared to the wild type (Figure 4E).

### Intracellular UGGT1 p.Arg1546^∗^ levels are diminished due to extracellular UGGT1 secretion

**Figure 5:**
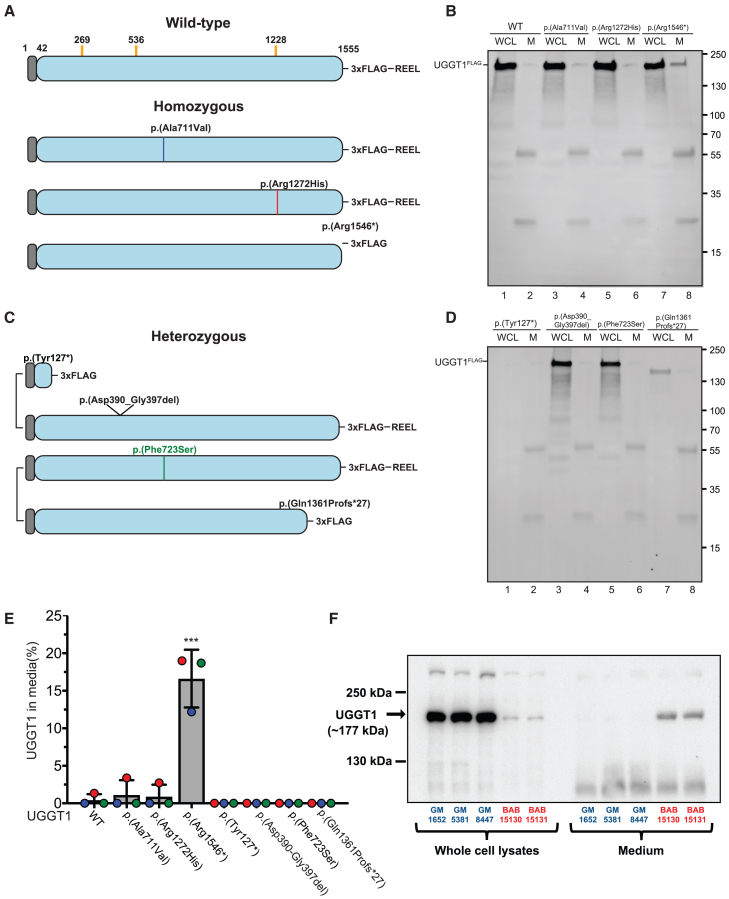
Molecular consequences of *UGGT1* variants identified in families with UGGT1-CDG (A) Description of wild-type (WT) and homozygous UGGT1 mutants. Gray indicates signal sequence cleaved upon entry into the ER, and blue denotes mature soluble protein. The orange line in the WT indicates sites of potential N-glycosylation sites found in UGGT1. All potential sites are preserved in all of the mutants except for the UGGT1 c.381_384del (p.Tyr127^∗^) mutant. A 3xFLAG tag was inserted before the C-terminal ER retrieval sequence (REEL) to allow for detection by western blot. *UGGT1* variants are noted with an asterisk, indicating the insertion of a premature stop codon. (B) Immunoblot measuring the expression and secretion of WT UGGT1 and homozygous UGGT1 mutants from (A) expressed in HEK cells. The levels of UGGT1 expressed within the cell (whole-cell lysate [WCL], lanes 1, 3, 5, and 7) was compared to levels secreted into the media (media [M], lanes 2, 4, 6, and 8) using a mouse monoclonal anti-FLAG antibody for immunoprecipitation. Detection was performed with an anti-FLAG polyclonal antibody. (C) Description of heterozygous UGGT1 mutants, similar to (A). The locations of the variants are similarly noted. (D) Immunoblot measuring expression and secretion of heterozygous UGGT1 mutants as described in (B). (E) Quantification of the levels of UGGT1 secretion into the media from (B) and (D). The percentage of UGGT1 in media was calculated by dividing the amount of UGGT1 quantified in the media by the total amount of protein in the WCL and media combined. Error bars represent standard deviation. ^∗∗∗^*p* < 0.001. Colored circles represent replicates. All western blots are representative of three independent biological replicates. (F) Western blot analysis of UGGT1 protein in WCL (left) and cell culture supernatants (right) from healthy control subjects’ fibroblasts (GM1652, GM5381, and GM8447, colored blue) and affected individuals with the homozygous UGGT1 variant p.Arg1546^∗^ (family 9: II-1 and II-2, labeled as BAB15130 and BAB15131, respectively, colored red), demonstrating a severe reduction in UGGT1 protein levels in patient WCLs with a concomitant increase in secreted UGGT1 protein (UGGT1 molecular weight = ∼177 kDa).

Since the recurrent *UGGT1* variant p.Arg1546^∗^ is predicted to remove ten C-terminal amino acids, including the ER retrieval sequence (REEL), but did not alter the *in vitro* or *in cellulo* glucosyltransferase activity, we hypothesized that the variant might impact subcellular localization and/or lead to its extracellular secretion. Therefore, we examined the consequences of *UGGT1* variants on intracellular protein levels by transfecting cells with FLAG-tagged wild-type or variant UGGT1 (Figures 5A–5E). With the exception of *UGGT1* p.Tyr127^∗^ and p.Gln1361Profs^∗^27, all *UGGT1* variants tested were robustly detected in WCLs (Figures 5B and 5D). While only trace amounts of wild-type and most variant UGGT1 were detected in cell culture media, a significant proportion of UGGT1 p.Arg1546^∗^ was secreted into media (Figures 5B and 5E).

We next examined UGGT1 protein levels in fibroblasts from two affected siblings in family 9 (individuals II:1 and II:2) and three unrelated healthy control subjects (Figure 5F). UGGT1 levels were markedly reduced in WCLs from both affected siblings in family 9 compared with healthy control subjects. To test the hypothesis that the observed reduction in intracellular UGGT1 might be due to its erroneous extracellular release, we incubated fibroblasts with a reduced volume of cell culture media for 3 days, filtered supernatants to remove cell debris, and examined the UGGT1 levels in media. While minimal UGGT1 was detected in supernatants from healthy control subjects’ fibroblasts, substantial amounts were found in supernatants from the affected individuals’ fibroblasts (family 9, II-1 and II-2) (Figure 5F). These findings suggest that the loss of the C-terminal ER retrieval sequence in *UGGT1* p.Arg1546^∗^ leads to its abnormal secretion, resulting in decreased intracellular UGGT1 levels (Figure 5F).

### Mini-gene splicing assay demonstrates that *UGGT1* variants disrupt *UGGT1* mRNA splicing

The *UGGT1* p.Ala711Val variant did not impact glucosylation in our cell-based assay or catalytic activity in our *in vitro* enzymatic assay. However, since the variant lies five nucleotides from the exon 19-intron 19 junction, we hypothesized that it might influence mRNA splicing. *In silico* predictions supported a splicing alteration (SpliceAI: donor loss, 0.79 and donor gain, 0.92; pangolin: splice loss, 0.75 and splice gain, 0.87). Therefore, we experimentally tested the impact of p.Ala711Val on mRNA splicing using a mini-gene assay.^28^ *UGGT1* p.Ala711Val was found to introduce a novel splice site within the exon, resulting in a 7-bp frameshift (Figure S6C). This result likely identifies aberrant splicing outcomes associated with the p.Ala711Val variant. We also tested the splicing impact of p.Arg1272His since it alters the last amino acid of exon 34 and is predicted to cause *UGGT1* splicing abnormalities (SpliceAI: acceptor loss, 0.58 and donor loss, 0.64; pangolin: splice loss, 0.69 and splice gain, 0.13). Moreover, similar to p.Ala711Val, the cell-based glucosylation assay did not show a significant impact on UGGT1 or AAT-Z glucosylation. The splicing assay showed that this variant results in alternative splicing, leading to both exon skipping and intron retention (Figure S5E).

## Discussion

Here, we present a clinical, genetic, and molecular delineation of a CDG (UGGT1-CDG) due to bi-allelic variants in *UGGT1*. UGGT1-CDG comprises a characteristic but variable multisystem phenotype entailing GDD and intellectual disability of ranging severity, including perinatal-childhood death. The cardinal clinical features include microcephaly, seizures, craniofacial dysmorphism, and behavioral abnormalities, including diagnoses of attention-deficit hyperactivity disorder (ADHD) and autism. More variable features of the condition include structural cardiac anomalies, including ventricular and ASDs; aortic arch anomalies and double outlet right ventricle; cryptorchism; renal anomalies (cystic/dysplastic kidneys); hepatomegaly; recurrent infections; and skeletal anomalies, such as scoliosis and/or vertebral anomalies. While cranial imaging appears normal in a proportion of affected individuals (4/14), there are nonspecific findings reported in most individuals that include subtle corpus callosum anomalies, periventricular heterotopia, cerebellar vermian hypoplasia/atrophy, and/or cerebral atrophy (Figure 3). It is possible that these findings are explained by perinatal hypoxia and/or comprise sequelae of the severe seizure presentations in affected individuals. Alternatively, a more characteristic UGGT1-CDG neuroimaging phenotype may emerge as further affected families are identified.

The clinical spectrum of UGGT1-CDG reflects that of other CDGs, which commonly cause multisystemic manifestations with developmental delay and neurological abnormalities of variable clinical severity.^4^ UGGT1-CDG displays notable clinical overlap with MOGS-CDG, in which glucosidase I enzyme function is disrupted several steps upstream of UGGT1 in the N-linked glycosylation quality control pathway. Clinical features common to both conditions include severe intellectual disability and GDD, seizures, congenital cardiac anomalies, hepatomegaly, hypoplastic genitalia, scoliosis, central apnea, craniofacial dysmorphology, and feeding problems (Table 1). While frequent infections and hypogammaglobulinemia are common in MOGS-CDG, recurrent infections were infrequently reported, and immunoglobulin levels have yet to be assessed in UGGT1-CDG. Although episodic neutropenia was reported in family 8, it is unclear whether this represents a rare feature of UGGT1-CDG or an unrecognized dual molecular diagnosis due to multilocus pathogenic variation within the family.^33^ Significant visual problems are commonly seen in MOGS-CDG and not infrequently noted in UGGT1-CDG, whereas sensorineural hearing loss, which is reported in a high proportion of MOGS-CDG, was observed only once in UGGT1-CDG. However, in-depth ophthalmological examination and audiological assessment has not been completed in all individuals with UGGT1-CDG, suggesting that some visual problems and hearing impairment may well be unrecognized. The majority of patients with UGGT1-CDG appear to have normal to increased muscle tone, although hypotonia in the neonatal period/infancy is seen, alongside the persistence of low central tone in some individuals. Interestingly, similar clinical features (specifically intellectual disability, dysmorphism, behavioral abnormalities, seizures, and variable liver dysfunction) are also observed in MAN1B1-CDG associated with defective mannosyl-oligosaccharide alpha-1,2-mannosidase function (also known as ERManI) encoded by *MAN1B1* (MIM: 604346).^10^^,^^34^ ER mannosidases can regulate UGGT1 activity by trimming the terminal mannose on the A-branch, creating a carbohydrate unable to be modified by UGGT1. This can occur after a set number of rounds in the CNX/CRT cycle^14^ (Figure 1).

UGGT1 encodes a 1,555-amino-acid protein with four N-terminal TRXLs thought to mediate the recognition of proteins with partial folding defects, a β sandwich region, and a C-terminal glucosyltransferase domain responsible for catalytic activity.^35^ Importantly, located at the extreme C terminus of UGGT1 is the ER retrieval sequence (REEL) motif, which is critical for the residence of the molecule within the ER.^36^ To investigate the impact of each variant on UGGT1 catalytic function, we performed complementary cell-based and *in vitro* catalytic activity assays, investigating UGGT1’s ability to reglucosylate both other proteins and itself. While the exact functional significance of this *self*-glucosylation mechanism remains unclear,^16^ it may serve as a mechanism for newly synthesized UGGT1 to enter the CNX/CRT cycle and achieve correct folding. Our assays confirmed that most variant *UGGT1* isoforms exhibit significantly reduced or near-absent glucosyltransferase activity (Figure 4). Although results were incongruent between the two assays for the p.Arg1272His variant and the p.Phe723Ser variant, with the *in vitro* assay showing a significant reduction in catalytic activity (*p* < 0.05), while the cell-based assay showed limited (p.Phe723Ser) to no (p.Arg1272His) impact on glucosyltransferase activity, this discrepancy could be due to overexpression of the mutant 3xFLAG UGGT1 in the cell-based assay. Furthermore, the difference between the effect of the p.Phe723Ser variant on *trans*-glucosylation and *self*-glucosylation observed in the cell-based assay is likely due to AAT-Z’s tendency to form large polymers within the ER, which may be unable to fit within the sensory domain of UGGT1 and thus remain unmodified.^37^

Three variants did not exhibit reduced glucosyltransferase activity (p.Ala711Val, p.Arg1272His, and p.Arg1546^∗^). Instead, our splicing assay showed that the p.Ala711Val variant caused a 7-bp exon truncation and frameshift in all of the mini-gene transcripts, and the p.Arg1272His variant caused retention of an intron in the majority of mini-gene transcripts, both likely resulting in a significant degree of UGGT1 loss of function via NMD (Figure S6). The p.Arg1546^∗^ variant introduces a premature stop codon within the penultimate *UGGT1* exon and is predicted to escape NMD and generate a polypeptide lacking the ER retrieval sequence, resulting in a significant reduction in intracellular retention of the UGGT1 (Figure 5). The p.Arg1546^∗^ variant is found in the heterozygous state in six individuals of non-Finnish European genetic ancestry (minor-allele frequency [MAF] = 5.4E−6), 1 individual of East Asian genetic ancestry (MAF = 2.52E−5), and absent in individuals of Middle Eastern genetic ancestry, although the total allele number within this ancestry is low (5,762 alleles) (gnomAD v.4.1.0). The shared haplotype surrounding the p.Arg1546^∗^ variant in affected individuals indicates that this most likely represents a rare clan genomics derived founder allele in Arab populations.^38^ The ultimate anticipated impact of all described variants is a disruption to the CRT/CNX cycle, resulting in the accumulation of misfolded glycoproteins within the ER, associated with ER stress, which has previously been observed in *UGGT1* knockout cells.^16^ It is likely that misfolded proteins are secreted from the ER and accumulate in other intra- and extracellular locations, which may contribute to cellular stress, dysregulated signaling, and immune activation, thereby exacerbating disease pathology. While this remains speculative, it highlights an additional avenue for exploring the mechanisms underlying UGGT1-CDG.

As with many monogenic conditions, the full clinical spectrum of disease becomes clearer as more affected individuals are identified and deeply phenotyped over time. Our current data suggest a fundamental correlation between disease severity and UGGT1 activity, as revealed by our enzymatic activity assay. Fetal demise/infantile death and the most severe multisystem phenotypes were seen in individuals with likely bi-allelic null alleles (i.e., likely loss-of-function variants). This includes family 3, homozygous for p.Ser327Phefs^∗^13, and family 1, compound heterozygous for p.Tyr127^∗^ and p.Asp390_Gly397del, the latter displaying nearly complete inactivation of glucosyltransferase activity. Notably, the fetal demise/early infant death observed in association with bi-allelic null alleles is less severe than the incomplete embryological lethality seen in knockout mice^17^ (see web resources [Mouse Genome Informatics UGGT1 mouse model]). This may be in part due to compensatory mechanisms in the CNX/CRT system that differ between humans and mice. In contrast, survival beyond infancy was seen in affected individuals harboring *UGGT1* missense variant(s) likely producing the UGGT1 enzyme, which is hypomorphic in nature and retains a degree of enzymatic activity (e.g., families 2 and 7 homozygous for p.Gln1155Arg and p.Arg1272His, respectively, or family 4 compound heterozygous for p.Gln1361Profs^∗^27 and p.Phe723Ser). Also of note, the recurrent *UGGT1* p.Arg1546^∗^ variant, which severely reduces but does not completely eliminate intracellular UGGT1 levels, is associated with survival beyond infancy and neurodevelopmental disabilities.

The primary molecular diagnostic test for N-linked glycosylation CDGs entails screening for abnormal transferrin glycosylation patterns in blood using isoelectric focusing. Historically, this method has been employed to categorize CDG subtypes into type I, which is associated with defects in N-linked glycan synthesis occurring in the cytoplasm or ER, and type II, which relates to further processing of the glycoprotein sugar chains typically within the Golgi apparatus.^4^^,^^39^ However, it was evident even before the emergence of molecular diagnostics that some CDGs do not exhibit transferrin glycosylation patterns that align with their molecular etiology. Consequently, the nomenclature has been revised to reflect these discrepancies.^4^ For example, in MOGS-CDG, where, despite the loss of glucosidase I enzymatic activity leading to unprocessed N-glycans (such as those found on transferrin), glycans are modified by endo-α-1,2-mannosidase. This modification results in a normal-appearing transferrin glycosylation pattern that masks the underlying molecular defect.^39^ CDGs are now defined according to the associated gene and categorized into four groups: N-linked, O-linked, lipid glycosylation defects, and glycosylphosphatidylinositol (GPI) anchor biosynthesis defects. Clinical transferrin analysis in individuals with UGGT1-CDG in our study (families 1, 2, and 8) displayed a normal transferrin pattern. While it has not been experimentally confirmed, the proximity of UGGT1 to glucosidase I in the N-linked glycosylation quality control pathway suggests the possibility of similar downstream effects, as both diminish the ability to engage the CNX/CRT cycle. This activity could modify transferrin and related glycoproteins, leading to normal glycosylation patterns. These findings suggest that transferrin analysis is not representative of the full spectrum of CDGs and that genetic testing should be performed upon strong clinical suspicion of a CDG regardless of transferrin patterning.

At present, treatment for CDGs is largely supportive; however, a number of targeted therapies for specific CDGs exist. These include mannose supplementation in CDG type Ib (MPI-CDG, MIM: 602579) and galactose supplementation in CDG type IIm (SLC35A2-CDG, MIM: 300896).^40^ Early diagnosis, which includes identifying the underlying genetic cause, is therefore essential to optimize outcomes for affected individuals.

Taken together, our data define bi-allelic *UGGT1* alterations as a genetic cause of a CDG with a severe multisystem phenotype, primarily impacting neurological function. Further studies are needed to understand the full phenotypic spectrum of UGGT1-CDG, genotype-phenotype correlations, and whether sugar supplementation might have therapeutic benefits. The identification of a 20-year-old male with UGGT1-CDG suggests that survival into adulthood is possible, but further case identification and follow-up are needed to establish the long-term prognosis for UGGT1-CDG.

## Data and code availability

All data described in this study are provided within the article and supplemental information. All ES and GS data generated by the BHCMG/BCM-GREGoR for which informed consent for deposition into controlled-access databases was provided were deposited into either dbGaP under BHCMG dbGaP study accession number phs000711.v7.p2 or the AnVIL repository under study name “Baylor-Hopkins Center for Mendelian Genomics” or “Baylor College of Medicine GREGoR” (https://anvilproject.org/).

## Acknowledgments

We dedicate this paper to the late Dan Hebert for his extraordinary contributions to UGGT science over the past 30 years. We are grateful to the families for participating in this study. This work was funded as follows: J.R.L., United States 10.13039/100000051National Human Genome Research Institute (NHGRI), the 10.13039/100000050National Heart, Lung, and Blood Institute (NHBLI) via the 10.13039/100016685Baylor-Hopkins Center for Mendelian Genomics, and the 10.13039/100000065National Institute of Neurological Disorders and Stroke (NINDS); C.G.S., A.H.C., and E.L.B., the 10.13039/100010269Wellcome Trust and the National Institute for Health and Care Research Exeter Biomedical Research Centre; J.E.P., J.R.L., and R.A.G., the NHGRI via the GREGoR Consortium and the Baylor College of Medicine Human Genome Sequencing Center; R.A.G., the Baylor College of Medicine Human Genome Sequencing Center; K.M.B., the GREGoR Consortium Research Grant from the GREGoR Data Coordinating Center, the National Eye Institute and Lions Foundation, and Research to Prevent Blindness Unrestricted Grant MEE; D.G.C., the Chao Physician Scientist Award, the United States 10.13039/100005202Muscular Dystrophy Association (MDA) Development Grant, and NINDS Child Neurologist Career Development Award; D.P., the Doris Duke Charitable Foundation, 10.13039/100000065NINDS, and the Blue Bird Circle Foundation; D.N.H., the NIH and a Chemistry-Biology Interface program training grant; R.M. and R.K., the Queen Square Brain Bank and the MRC Brain Bank Network; and H.H.F. and B.G.N., The Rocket Fund and the NIH. This work was also funded by the 10.13039/100009633Eunice Kennedy Shriver National Institute of Child Health & Human Development of the NIH and the Science and Technology Development Fund (STDF), Academy of Science Research and Technology, Egypt. See the supplemental information for full acknowledgments and grant reference numbers where applicable.

## Author contributions

E.L.B., A.H.C., and J.R.L. conceived and supervised the study. Y.T., H.H.F., and D.N.H. supervised the enzymatic assays and cellular work. Z.D., L.H., D.G.C., C.G.S., B.G.N., A.K.S., H.D., R.S., S.G.P., S.N.J., S.G., M.S.A.-H., M.K.H.A., R.M., R.K., K. Salayev, W.D.J., A.P.C., L.M., M.D., N.W., M.S., L.O., P.G., A.M.N., A.B., S.F.A.-G., M.S.Z., H.V.E., J.E.P., O.K.W., E.M.S., K.M.B., R.A.G., D.P., D.M., J.S.L., N.U., J.D., M.O., S.B., A.T., L.A., and F.S.A. performed genetic and clinical studies/analyses. T.K., K.P.G., and K. Sano performed and/or analyzed the cell and enzymatic studies. E.L.B., A.H.C., D.G.C., J.S.L., H.H.F., and J.R.L. coordinated/managed collaborations. Z.D., L.H., D.G.C., C.G.S., Y.T., H.H.F., D.N.H., J.R.L., A.H.C., and E.L.B. wrote the initial draft of the manuscript.

## Declaration of interests

J.R.L. is a consultant for Genome International. A.B. is an employee of and may hold stock in GeneDx, LLC. D.P. provides paid consulting services to Ionis Pharmaceuticals.

## Web resources

Anabaptist Variant Server (AVS), https://edn.som.umaryland.edu/Anabaptist/query.htm
Congenital disorders of N-linked glycosylation and multiple pathway overview, GeneReviews, https://www.ncbi.nlm.nih.gov/books/NBK1332/
GenBank, https://www.ncbi.nlm.nih.gov/genbank/
gnomAD (Genome Aggregation Database), https://gnomad.broadinstitute.org/
Mouse Genome Informatics UGGT1 mouse model, https://www.informatics.jax.org/marker/MGI:2443162
National Library of Medicine ClinVar, https://www.ncbi.nlm.nih.gov/clinvar/
OMIM, http://www.omim.org
PMM2-CDG GeneReviews, https://www.ncbi.nlm.nih.gov/books/NBK1110/

## Supplemental information

Data S1. Table S1

Document S2. Article plus supplemental information
